# Microfluidic Design of Streamlined Alginate Hydrogel Micromotors with Run and Tumble Motion Patterns

**DOI:** 10.1002/advs.202304995

**Published:** 2023-10-12

**Authors:** Jiabin Luan, Peter F. Kuijken, Wen Chen, Danni Wang, Levy A. Charleston, Daniela A. Wilson

**Affiliations:** ^1^ Radboud University Nijmegen Institute for Molecules and Materials Heyendaalseweg 135 Nijmegen 6525 AJ The Netherlands

**Keywords:** alginate hydrogel, microfluidics, micromotors, motion patterns, streamlined morphology

## Abstract

Autonomous micromotors demonstrate remarkable advancements in biomedical applications. A noteworthy example is streamlined motors, which display enhanced movement efficiency with low fluid‐resistance. However, existing streamlined motors, primarily constructed from inorganic materials, present challenges due to their complex fabrication procedures and lack of a soft interface for interaction with biological systems. Herein, a novel design of biodegradable streamlined alginate hydrogel micromotors with a teardrop shape by microfluidics is introduced. The platform enables the high‐throughput fabrication of monodisperse micromotors with varied dimensions. By incorporating Pt‐coated Fe_3_O_4_ nanoparticles, micromotors are equipped with dual capabilities of catalytic propulsion and accurate magnetic guidance. Through precisely tuning the localization regions of catalysts within the micromotors, the streamlined hydrogel micromotors not only exhibit enhanced propelling efficiency, but also accomplish distinct motion patterns of run and tumble. The design provides insights for developing advanced micromotors capable of executing intricate tasks across diverse application scenarios.

## Graphical abstract

Streamlined alginate hydrogel micromotors with a teardrop shape by microfluidics are generated by microfluidics, enabling high‐throughput fabrication of monodisperse micromotors with precisely varied dimensions. Tuning the localization regions of catalysts within the streamlined hydrogel micromotors enhances propelling efficiency and accomplishes distinct motion patterns of run and tumble.

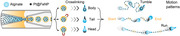

## 1 Introduction

Advanced micromotors with autonomous movement by harnessing energy have the ability to accomplish intricate tasks that surpass the capabilities of traditional passive systems.^[^ ^1^, ^2^ ^]^ Proof‐of‐concept demonstrations have been employed in in vivo imaging,^[^ ^3^ ^]^ targeted delivery,^[^ ^4^ ^]^ environmental remediation,^[^ ^5^ ^]^ and artificial communication.^[^ ^6^, ^7^ ^]^ To date, micromotors with diverse architectures have been fabricated in various shapes, such as rods,^[^ ^8^ ^]^ tubes,^[^ ^9^, ^10^ ^]^ spheres,^[^ ^3^, ^11^ ^]^ and stomatocytes.^[^ ^12^, ^13^ ^]^ Inspired by the efficient examples in nature, such as fishes, tadpoles, and sperms, motors with a streamlined shape have demonstrated superior potential because of their enhanced movement efficiency resulting from reduced moving resistance.^[^ ^14^, ^15^, ^16^, ^17^ ^]^ Unfortunately, the streamlined motors reported thus far have predominantly been constructed using inorganic silica, which lacks a soft interface for favorable biocompatibility in biomedical applications.^[^ ^18^, ^19^, ^20^ ^]^ Furthermore, an essential element for the active motors is the asymmetrical distribution of catalysts.^[^ ^21^ ^]^ However, the formation of the streamlined silica motors requires either appropriate templates or complex growth steps,^[^ ^14^, ^15^, ^16^ ^]^ which are labor‐intensive and present challenges in efficiently incorporating catalysts into the microparticles. Therefore, the high‐throughput fabrication of soft and biodegradable streamlined micromotors is of primary importance and is urgently desired. The streamlined motors provide a viable solution to enhance the speed of motors. An equally significant but often overlooked aspect lies in the exploration of motion patterns for motors. Bacteria provide a captivating illustration in nature as they adaptively regulate their motion patterns, alternating between runs and tumbles during chemotaxis.^[^ ^22^, ^23^, ^24^ ^]^ Distinct motion patterns in motors are imperative for propelling and executing complex tasks across diverse application scenarios.

Microfluidics has emerged as a powerful platform for generating monodisperse microscale droplets with a high throughput.^[^ ^25^, ^26^, ^27^, ^28^ ^]^ Through the incorporation of functional elements, micromotors with systematically varied dimensions, structures, and compositions can be fabricated precisely by microfluidics.^[^ ^12^, ^29^, ^30^, ^31^ ^]^ In this study, we show the first design of biodegradable streamlined alginate hydrogel micromotors by microfluidics. A streamlined teardrop shape was achieved by the spontaneous separation and cross‐linking of alginate drops from the double emulsion templates as generated by microfluidics. Notably, the design adopts a facile and mild cross‐linking method using Ca^2+^ ions. After loading catalysts, the inherent asymmetry of the teardrop shape is expected to achieve self‐propulsion because of the asymmetrical distribution of the catalysts. Furthermore, we made attempts to regulate the localization of the catalysts within the micromotors. We hypothesized that by locating the catalysts in different regions of the micromotors, it would be feasible to achieve different motion patterns. To test our hypothesis, we synthesized platinum‐coated iron oxide nanoparticles (Pt@FeNPs) as the model catalyst for the micromotors. The Pt@FeNPs offer a catalytic capacity, where Pt decomposes H_2_O_2_ for bubble propulsion, along with a magnetic response resulting from Fe_3_O_4_ when subjected to magnetic fields. By applying a weak magnetic field during the cross‐linking, we could control the localization of the Pt@FeNPs in different regions of the teardrop‐shaped particles. We envision that these various types of streamlined micromotors can accomplish distinct motion patterns when the fuel of H_2_O_2_ is introduced.

## 2 Results and Discussion

**Figure 1:**
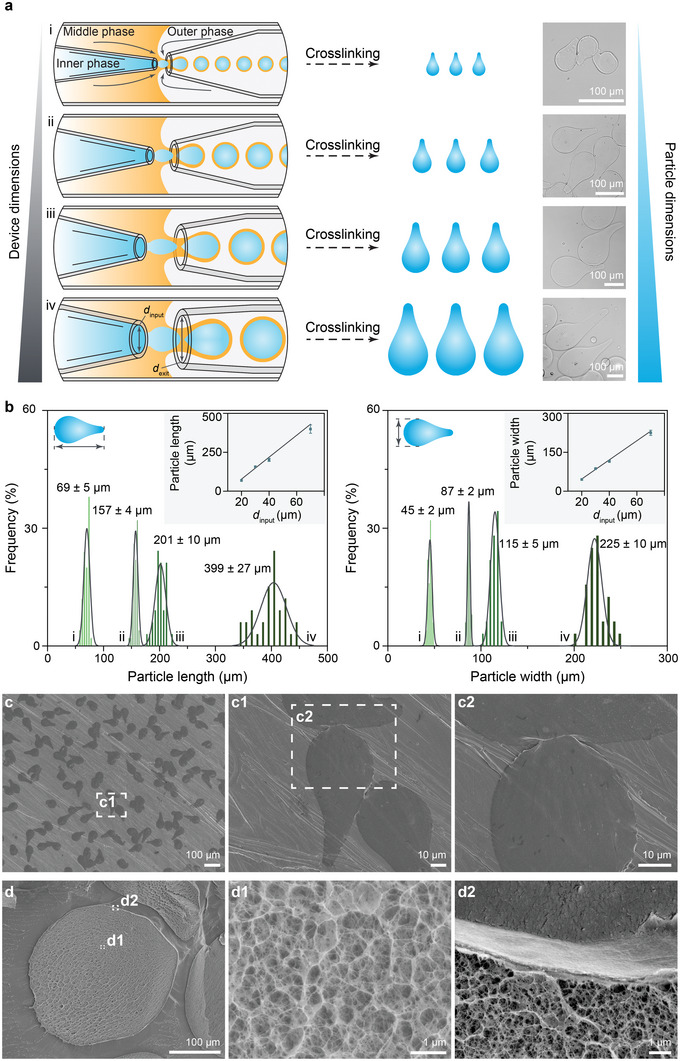
Fabrication and characterization of teardrop‐shaped alginate hydrogel microparticles. a) Generation of particles with various sizes from devices with different dimensions. Diameters of the input (*d*_input_) and exit (*d*_exit_) capillaries of devices i), ii), iii), and iv) (*d*_input_−*d*_exit_) are 20−40, 30−60, 40−80, and 70−110 µm, respectively. The formation of teardrop‐shaped hydrogel particles was imaged by bright field microscope. b) Frequency histograms showing the particle length and width of teardrop‐shaped alginate hydrogel particles with a high monodispersity as generated by devices i, ii, iii, and iv with different dimensions in (a) (n = 50 for each device). Histograms were fitted with Gaussian distributions (solid lines), and the data is presented as mean ± standard deviation. Hydrogel particle length and width are shown in the insets relative to the diameter of the input capillary (*d*_input_). Error bars indicate standard deviation, and lines represent the linear fit. c) SEM images of teardrop‐shaped alginate hydrogel particles. d) Cryo‐SEM images of the cross‐section of teardrop‐shaped alginate hydrogel particles. (c1, c2) and (d1, d2) are magnified images of the corresponding regions in (c, c1) and (d), respectively.

Streamlined teardrop‐shaped hydrogel microparticles were efficiently fabricated using glass microfluidic devices, ensuring high‐throughput production and uniformity.^[^ ^32^ ^]^ Double emulsion droplets consisting of the alginate core (Inner phase) and mineral oil shell (Middle phase) were first generated by coaxial continuous phases (Outer phase) (**Figure** **1**a; Experimental Section and Figure S1a, Supporting Information). The double emulsion droplets were collected in 0.1 m CaCl_2_ solution. Because of the higher density of the alginate core phase, the alginate solution progressively ruptured the oil shell, initiating the physical cross‐linking upon contact with Ca^2+^ in the collection solution. The alginate droplets were continuously squeezed out due to the line tension between the oil and alginate phases, while their teardrop shape was formed due to the comparable interfacial tension between the two aqueous phases (Figure S2, Supporting Information).^[^ ^32^ ^]^ Different from the fluids with low viscosities, the high viscosity of alginate reduces the focusing capacity of the outer flow, resulting in a weak dependence between the droplet size and the flow speed of the outer flow.^[^ ^33^ ^]^ Alternatively, the dimensions of the microfluidic devices, that is the diameters of the input (*d*_input_) and exit (*d*_exit_) capillaries of device, allow the fine control of the particle sizes (Figure 1a). We can fabricate significantly larger teardrop‐shaped hydrogel particles by increasing the dimensions of devices, as demonstrated by the measurement of the length and width of particles (Figure 1b). Specifically, the particle dimensions averagely increase from 70 to 400 µm in length and 45 to 225 µm in width by using devices with larger dimensions. We obtained a linear relationship between the particle length and width and the *d*_input_ of devices (insets in Figure 1b). Each device yielded a narrow and monodisperse size distribution of particles, with coefficients of variation (CV%) ranging from 3 to 7% for length and from 2 to 4% for width, relative to their respective means. The hydrogel particles display a rather smooth feature, as depicted in the scanning electron microscopy (SEM) images (Figure 1c). Cryo‐scanning electron microscopy (cryo‐SEM) further revealed the characteristic internal porous structures of the hydrogel (Figure 1d,d1). The presence of the capsule‐like surface suggests a higher degree of cross‐linking, which can be attributed to the easy accessibility of Ca^2+^ ions (Figure 1d2).^[^ ^34^ ^]^ Therefore, a gradient of decreased cross‐linking degrees would be expected from the surface to the inner region of the hydrogel particles.

**Figure 2:**
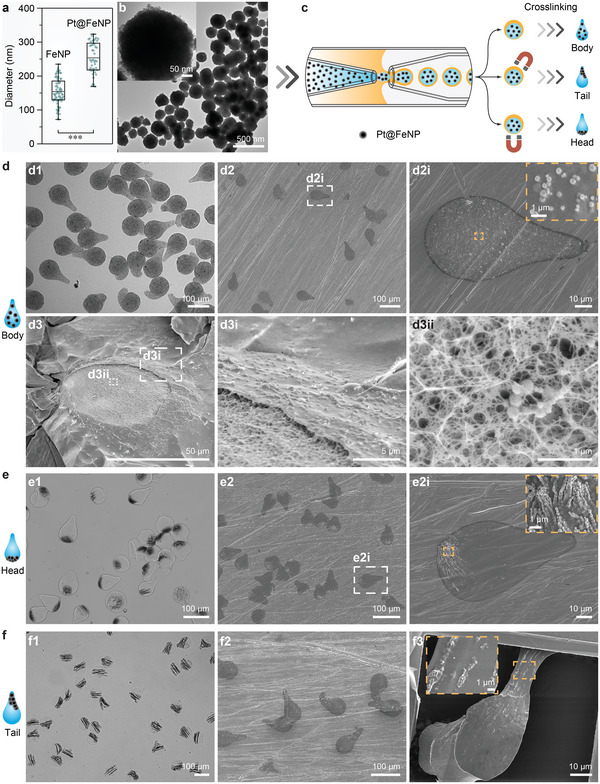
a) Diameter distribution of FeNPs and Pt@FeNPs (n = 50). ^***^ *p*<0.001; an unpaired *t*‐test was used. b) TEM image of Pt@FeNPs. An individual Pt@FeNP is shown in the inset. c) Generation of Pt@FeNPs loaded particles (10 mg mL^−1^), followed by the control of the Pt@FeNPs distributions via magnet during cross‐linking. d) Characterization of evenly Body distributed particles: (d1) bright field microscopic image; (d2, d2i) SEM images; (d3, d3i, d3ii) cryo‐SEM images. (d2i) and (d3i, d3ii) are magnified images of the corresponding regions in (d2) and (d3), respectively. A magnified region is shown in the inset of (d2i). e) Characterization of head shifted particles: (e1) bright field microscopic image; (e2, e2i) SEM images. (e2i) is a magnified image of the corresponding region in (e2). A magnified region is shown in the inset of (e2i). f) Characterization of tail shifted particles: (f1) bright field microscopic image; (f2) and (f3) SEM images. A magnified region is shown in the inset of (f3).

To design hydrogel micromotors with various accumulation sites of catalysts, we synthesized Pt@FeNPs as the model catalyst, which is capable of both magnetic guidance and chemical activity. FeNPs with a diameter of 150 nm were first synthesized, followed by the synthesis of Pt on the surface of the FeNPs (Pt@FeNPs). The diameter of resulting Pt@FeNPs significantly increased to ≈250 nm because of the formation of Pt on FeNPs (**Figure** **2**a,b; Figure S3, Supporting Information). The concentrations of Pt and Fe in Pt@FeNPs were determined to be 54 and 34 wt.%, respectively, using inductively coupled plasma mass spectroscopy. Microfluidics allowed a facile encapsulation of substances of interest by mixing them with inner phase before the generation of double emulsion droplets (Figure 2c). By mixing Pt@FeNPs with the alginate solution and the same cross‐linking procedure, we obtained nearly evenly body distributed particles (Body), as demonstrated by the bright field image (Figure 2d1). Interestingly, we observed that a majority of the encapsulated Pt@FeNPs were situated at the interface of the droplet and on the surface of the hydrogel particles, resulting in surface roughness when compared to the smooth surface of empty hydrogel particles (Figure 2d2i,d3i). Some encapsulated Pt@FeNPs could be occasionally encountered inside the porous network of hydrogel (Figure 2d3ii). The accumulation of nanoparticles toward the surface of alginate is driven by the reduced interfacial energy in the double emulsion droplets.^[^ ^11^ ^]^ We expect the surface roughness would facilitate the pinning of oxygen bubbles when the micromotors were exposed to the chemical fuel of H_2_O_2_.^[^ ^12^ ^]^ This could potentially enable the achievement of highly efficient motors with high speeds at low concentration of fuel. To further control the localization of the Pt@FeNPs within the hydrogel particles, we positioned a magnet either at the bottom or top of the collection vial during the cross‐linking procedure (Figure 2c). The Pt@FeNPs were aligned in chains under a magnetic field of ≈230 mT, and migrated either to the bottom or top of the alginate cores depending on the location of the magnet. Upon the completion of cross‐linking, the Pt@FeNPs were trapped within the network of hydrogel, resulting in the formation of either head shifted (Head) or tail shifted (Tail) particles (Figure 2e,f).

**Figure 3:**
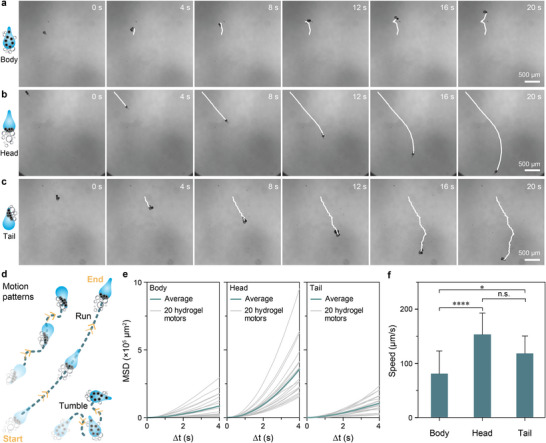
Motion patterns investigation of hydrogel micromotors. a−c) Motion trajectories of Body, Head, and Tail hydrogel micromotors in 3.5% H_2_O_2_. d) Schematic motion patterns of Body, Head, and Tail hydrogel micromotors. e) MSD curves of 20 individual (gray) and averaged (green) Body, Head, and Tail hydrogel micromotors in 3.5% H_2_O_2_. f) Speeds of Body, Head, and Tail hydrogel micromotors in 3.5% H_2_O_2_. The significance tests were conducted using Kruskal–Wallis tests. n.s. = 0.122, ^*^ *p* value < 0.05, ^****^ *p* value < 0.0001.

Next, we explored the possibility of achieving distinct motion patterns among the three types of motors by the addition of 3.5% H_2_O_2_ as the chemical fuel. As expected, Body hydrogel micromotors consistently displayed multiple bubble pinning positions because of the evenly body distributed Pt@FeNPs, resulting in a tumbling‐type motion trajectory (Tumble) (**Figure** **3**a; Movie S1, Supporting Information). In contrast, Head and Tail hydrogel micromotors exhibited more linear motion trajectories (Run), with the motors moving predominantly toward their respective tail and head direction (Figure 3b,c; Movies S2 and S3, Supporting Information). Such remarkable behaviors are a consequence of the bubble pining occurring in the head and tail regions, where the catalysts are localized. Interestingly, the Tail micromotors also showed occasional circulation motion with rotation (Movie S4, Supporting Information). This is attributed to the repeated preferential bubble pining on one side of the tail, leading to a constant propulsion angle of motors.^[^ ^12^ ^]^ The different motion patterns of the Body, Head, and Tail micromotors are schematically depicted in Figure 3d. To compare the speeds of the different hydrogel micromotors, twenty of the most efficient motors for each type were tracked for 20 s in 3.5% H_2_O_2_. It is noteworthy that no surfactant was used in our motion studies, which is often used to facilitate the generation of bubbles for propulsion.^[^ ^9^, ^11^, ^35^ ^]^ Mean squared displacement (MSD) curves up to 4 s for each type of motor were obtained from the trajectories in the 20 s motion movies (Figure 3e). The resulting averaged MSD curves display a parabolic feature, indicating ballistic motion of the hydrogel micromotors. The speeds of the three types of micromotors were obtained from the trajectories (Figure 3f). The Head micromotors exhibited the highest propulsion speed, primarily due to the localization of catalysts in the head region, which facilitated bubble pinning in the same area. The speed of the Tail micromotors was influenced by the occasional rotational motion, while the Body micromotors propelled the least efficiently because of their dispersed distribution of catalysts and multiple bubble pinning locations.

**Figure 4:**
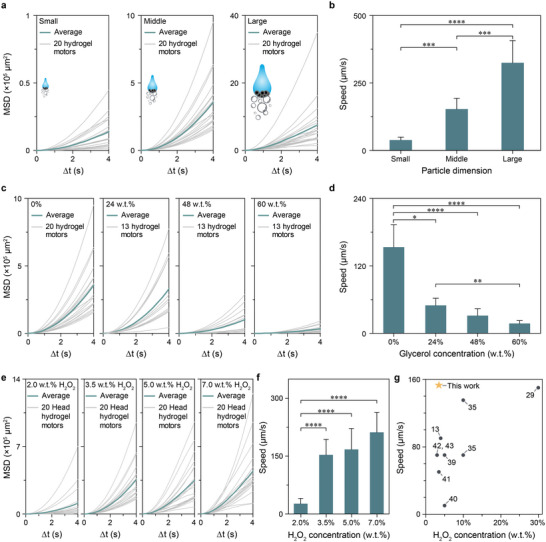
Motion speeds investigation of Head hydrogel micromotors. a) MSD curves of 20 individual (gray) and averaged (green) Small, Middle, and Large hydrogel micromotors in 3.5% H_2_O_2_. b) Speeds of Small, Middle, and Large hydrogel micromotors in 3.5% H_2_O_2_. Small, Middle, and Large refer to particles produced by devices i, ii, and iv, respectively, with input‐output diameters (*d*_input_−*d*_exit_) of 20−40, 30−60, and 70−110 µm. c) MSD curves of individual (gray) and averaged (green) Middle hydrogel micromotors in different glycerol concentrations with 3.5% H_2_O_2_. d) Speeds of Middle hydrogel micromotors as a function of glycerol concentrations with 3.5% H_2_O_2_. e) MSD curves of 20 individual (gray) and averaged (green) Middle hydrogel micromotors in different H_2_O_2_ concentrations. f) Speeds of Middle hydrogel micromotors as a function of H_2_O_2_ concentrations. g) Comparison of motion speeds between this work and others in the literature. Numbers indicate the corresponding cited references. Statistical analyses were performed using Kruskal–Wallis tests. ^*^ *p* value < 0.05, ^**^ *p* value < 0.01, ^***^ *p* value < 0.001, ^****^ *p* value < 0.0001.

To investigate the relation between the motion speed versus particle dimensions, viscosity, and fuel concentration, Head hydrogel micromotors were selected as the model system. Using devices with varying dimensions (Figure 1a), Small, Middle, and Large micromotors could be generated. The average MSDs and speeds of micromotors exhibited a significant increase in correlation with the progressive augmentation of their dimensions (**Figure** **4**a,b). The enhanced motion capability can be attributed to the substantially higher loading of catalysts within particles of larger dimensions, while maintaining the same catalyst concentration. To explore the relationship between motion speed and viscosity, the viscosity of the solution was varied from 1.0 mPa • s (water, 0% glycerol) to 10.9 mPa • s (60% glycerol) by dissolving glycerol to water to simulate in vivo environment (Table S1, Supporting Information). The concentration of H_2_O_2_ fuel remained constant at 3.5% throughout the study. Upon increasing the glycerol concentration from 0% to 24%, the average speed of micromotors decreased from 150 to 50 µm s^−1^. The speed further decreased to 18 µm s^−1^ as the glycerol concentration increased to 60% (Figure 4c,d). Furthermore, we noticed an increase in bubble size along with a decreased bubble release frequency in the presence of glycerol. The improved bubble stability could potentially stem from the prevention of evaporation by glycerol.^[^ ^36^ ^]^ The decrease in propulsion velocity can be ascribed to both the increased drag force of the medium due to higher viscosity and a decreased frequency of bubble release.^[^ ^37^, ^38^ ^]^ The average MSDs and speeds of the micromotors were dependent on the concentration of the chemical fuel (Figure 4e,f). The motors exhibited a relatively slow speed of 27 µm s^−1^ in 2.0% H_2_O_2_, which significantly increased to 150 µm s^−1^ in 3.5% H_2_O_2_. The substantial increase in speed suggests the presence of a threshold concentration of H_2_O_2_ that enables the efficient generation of oxygen bubbles to propel the micromotors. Further increasing the concentration of H_2_O_2_ gradually enhanced the speed of the motors, reaching a value of 210 µm s^−1^ in 7.0% H_2_O_2_. Compared to other motors of similar sizes (larger than 100 µm) that also utilized Pt as the catalyst,^[^ ^13^, ^29^, ^35^, ^39^, ^40^, ^41^, ^42^, ^43^ ^]^ our Head hydrogel motors were more efficient with higher propelling speeds at a relatively low concentration of 3.5% H_2_O_2_ (Figure 4g). We speculated the localization of catalysts, along with the surface roughness and porous network of alginate hydrogel, played significant roles in facilitating the efficient generation and pinning of oxygen bubbles. These factors together with the streamlined shape collectively contributed to the enhanced performance and efficiency of the Head micromotors. Although current micromotors are constructed from biocompatible alginate and exhibit degradation properties (Figure S4, Supporting Information), the use of H_2_O_2_ in large concentrations as fuel is not biocompatible. Therefore, alternative fuel sources should be explored in forthcoming research endeavors. For example, instead of using Pt@Fe NPs as catalysts, magnesium‐based magnetic particles could employ water and acid as the fuel to drive the propulsion of micromotors.^[^ ^44^, ^45^ ^]^

**Figure 5:**
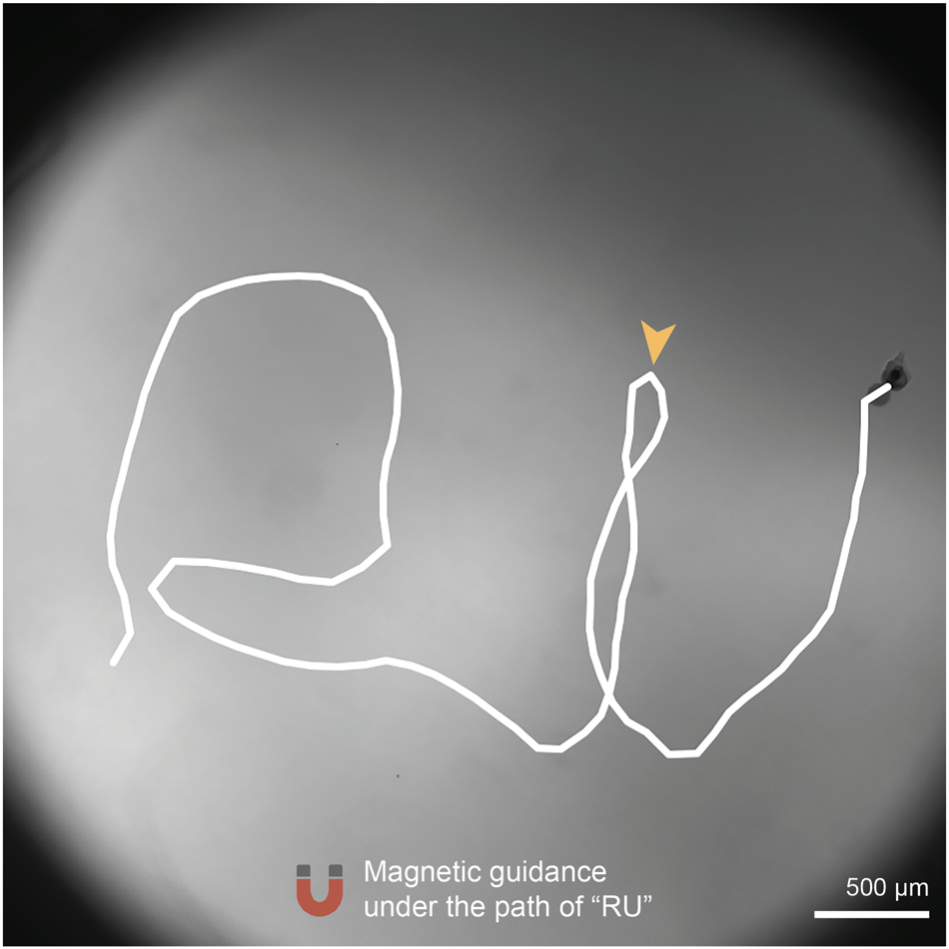
Magnetic guidance of the Head hydrogel micromotor under the path of “RU”.

Finally, as a proof‐of‐concept, we demonstrated magnetic guidance of the Head micromotors in 3.5% H_2_O_2_. A ≈0.1 mT magnetic field was applied to guide the micromotor in a predescribed and challenging path that follows “RU” (**Figure** **5**; Movie S5, Supporting Information). The motor demonstrated directional accuracy when subjected to magnetic steering, as evidenced by its remarkable U‐turn capability (arrow in Figure 5). This implies that the motors can be precisely maneuvered toward the targeting location.

## 3 Conclusion

In summary, we have developed a high‐throughput microfluidic design for the fabrication of streamlined teardrop‐shaped hydrogel micromotors. The dimensions of the alginate micromotors could be precisely adjusted by tuning the sizes of the microfluidic devices. Three types of micromotors (Head, Body, and Tail), as characterized by the various distribution of catalysts, were obtained, which exhibited distinct motion patterns. The current microfluidic design provides a versatile platform that integrates crucial features for the next‐generation micromotors, including low‐cost fabrication, high‐throughput production with precise control, biodegradability, high propelling efficiency, and accurate magnetic guidance. We envision that the present micromotors hold great potential, particularly in biomedical applications. Future investigations are necessary to demonstrate the capability for executing intricate tasks in applications such as target drugs^[^ ^46^ ^]^ and cell delivery.^[^ ^32^ ^]^

## Conflict of Interest

The authors declare no conflict of interest.

## Supporting information

Supporting Information

Supplemental Movie 1

Supplemental Movie 2

Supplemental Movie 3

Supplemental Movie 4

Supplemental Movie 5

## Data Availability

The data that support the findings of this study are available from the corresponding author upon reasonable request.
