# Supplementary material for: Microfluidic Design of Streamlined Alginate Hydrogel Micromotors with Run and Tumble Motion Patterns
Source: Adv Sci (Weinh). 2023 Oct 12;10(34):2304995. doi: 10.1002/advs.202304995 (PMC10700165; doi:10.1002/advs.202304995)
Supplement: Supplementary file 1 — Supporting Information [file ADVS-10-2304995-s002.pdf]

ADVANCED SCIENCE
Open Access

Supporting Information

for *Adv. Sci.*, DOI 10.1002/advs.202304995

Microfluidic Design of Streamlined Alginate Hydrogel Micromotors with Run and Tumble Motion Patterns

*Jiabin Luan, Peter F. Kuijken, Wen Chen, Danni Wang, Levy A. Charleston and Daniela A. Wilson**

Supporting Information

Microfluidic Design of Streamlined Alginate Hydrogel Micromotors with Run and Tumble Motion Patterns

Jiabin Luan, Peter F. Kuijken, Wen Chen, Danni Wang, Levy A. Charleston, and Daniela A. Wilson*

Radboud University, Institute for Molecules and Materials, Heyendaalseweg 135, 6525 AJ, Nijmegen, The Netherlands

*Corresponding author. e-mail: d.wilson@science.ru.nl

Table of Contents

S1 | Chemicals 3
S2 | Experimental: methods 4
S2.1 | Fabrication of microfluidic devices 4
S2.2 | Microfluidic and collection solutions 4
S2.3 | Microfluidic generation of monodisperse droplets 4
S2.4 | Collection, cross-linking and washing of hydrogel particles 5
S2.5 | Synthesis of Fe_3O_4 nanoparticles (FeNPs) 5
S2.6 | Synthesis of platinum-coated Fe_3O_4 nanoparticles (Pt@FeNPs) 5
S2.7 | Fabrication of hydrogel particles with various Pt@FeNPs localization 6
S2.8 | Inductively coupled plasma mass spectroscopy (ICP-MS) measurements 6
S2.9 | Characterization of surface and internal structures of hydrogel particles 6
S2.10 | Autonomous motion analysis 7
S2.11 | Statistical analysis 7
S3 | Supplementary Figures 8
S4 | References 14

S1 | Chemicals

Sodium alginate (Algin IL-1, viscosity of 1% solution: 10~20 mPa·s) was a kind gift from Kimica Corporation. Span 80, tween 20, light mineral oil, glycerol, Pluronic P123, Pluronic F127, trimethylsiyl chloride, 1H,1H,2H,2H-perfluoro-1-octanol, sodium acetate trihydrate ($NaOAc \cdot 3H_2O$), potassium tetrachloroplatinate (K_2PtCl_4) and were all purchased from Sigma-Aldrich. Calcium chloride ($CaCl_2$) was a product from Fisher Scientific. Iron(III) chloride hexahydrate ($FeCl_3 \cdot 6H_2O$) was a product from Merck. Heptane was purchased from VWR International B.V. Ethylene glycol was a product from Acros. Ascorbic acid was purchased from Fluorochem. Hydrogen peroxide (H_2O_2, 35%) was from J.T. Baker. Milli-Q (MQ) water was filtered through 0.22 μm syringe filter before usage. Simulated gastric fluid was prepared according to the description on Sigma-Aldrich (pH 1.1−1.3, ≈2.0 g L^{-1} sodium chloride and ≈2.917 g L^{-1} HCl).

S2 | Experimental: methods

S2.1 | Fabrication of microfluidic devices

Fabrication of microfluidic devices for creating monodisperse double emulsion droplets was adapted from previous literatures.[1-2] Cylindrical glass capillaries (inner diameter: 300 µm, outer diameter: 1 mm; Hilgenberg) were heated and pulled by a capillary puller (PN-31, Narishige) to form tapered capillaries. The tapered glass capillaries were then polished to the desired orifice diameters with sandpaper. The diameters were determined using a forge station (Microforge, MF 830, Narishige). Typical diameters of the capillaries were in the range of 20 to 70 µm for input (d_{input}), and 40 to 110 µm for exit (d_{exit}) capillaries, respectively. The input capillaries were treated with trimethylsiyl chloride to render them hydrophobicity. The input and exit capillaries were aligned coaxially inside a square capillary (inner diameter: 1 mm, Vitrocom). Three 25G dispensing needles (Henke Sass Wolf) were sealed with a two-component epoxy glue (Bison) at the junctions, serving as inlets for the fluids.

S2.2 | Microfluidic and collection solutions

To generate alginate hydrogel particles from double emulsion droplets, three different solutions and the collection solution were prepared according to the previous report.[1]

Inner phase was prepared by dissolving 2 wt.% sodium alginate in MQ water. As-prepared solution could be used for about one week.

Middle phase (oil phase) consisted of 0.4 wt.% Span 80 in light mineral oil. This solution remains stable for several weeks.

Outer phase was prepared by dissolving 0.5 wt.% Tween 20 in the mixture of 40 wt.% of glycerol and 59.5 wt.% of MQ water. As-prepared solution could last for weeks.

Collection phase (cross-linking solution) consisted of 0.1 M aqueous solution of $CaCl_2$ with 2 wt.% Pluronic P123 to increase the stability of mineral oil drops in the collection phase. The solution can be used for weeks.

S2.3 | Microfluidic generation of monodisperse droplets

The three solutions (inner, middle, and outer phases) were loaded into three Luer Lock syringes. The syringes were connected to the inlets of device using polytetrafluoroethylene tubing (inner diameter: 0.50 mm, outer diameter: 1.00 mm, wall thickness 0.25 mm; Bohlender). The fluid

flow rates were controlled with syringe pumps (Chemyx). Droplet formation was visualized in an inverted microscope (Axiovert 135 TV, Zeiss) with a camera (GS3-U3-23S6M-C, Flir). The flow rates of the three solutions were adjusted carefully to generate double emulsion droplets.

S2.4 | Collection, cross-linking and washing of hydrogel particles

The double emulsion droplets were collected in an 1.5 mL Eppendorf vial containing collection phase. Because of the higher density of the inner phase than the surrounding oil[1], the inner drop gradually comes in contact with the $CaCl_2$ solution. Cross-linking was immediately initiated, resulting in the formation of physically crosslinked hydrogel. The teardrop shape of hydrogel particles was a result of the low interfacial tension between the two aqueous fluids. The microparticles were left undisturbed for 10 minutes followed by centrifuging for one minute at 2000 rpm. The excess $CaCl_2$ solution was removed and 100 μL MQ water was added. Subsequently, 40 μL of 1H,1H,2H,2H-perfluoro-1-octanol and 160 μL heptane were added to the mixture. The mixture was pipetted thoroughly. After standing for seconds, the solution separated into three phases consisting of a top layer of heptane, middle aqueous layer with the microparticles, and bottom layer of 1H,1H,2H,2H-perfluoro-1-octanol. The aqueous phase with the microparticles was collected and washed with MQ water for five times (5 × 1.5 mL). The microparticles were re-dispersed in MQ water for future use. Noteworthily, the washing steps needed to be performed thoroughly to remove any surfactant from previous steps. Otherwise, the remaining surfactant may stabilize bubbles in the motion studies, which could cause interference.

S2.5 | Synthesis of Fe_3O_4 nanoparticles (FeNPs)

Iron oxide nanoparticles (FeNPs) were synthesized according to a previous report.[3] Briefly, $FeCl_3 \cdot 6H_2O$ (2.7 g) and $NaOAc \cdot 3H_2O$ (3.1 g) were added to 150 mL of ethylene glycol, followed by vigorous magnetic stirring for 30 min. The solution was transferred into a Teflon-lined stainless steel autoclave and heated in an oven at 200 °C for 12 hours. Subsequently, the particles were separated from solvent by placing a magnet under the vial and washed with water and ethanol for three times. Collected particles were dried under vacuum and grinded for further use.

S2.6 | Synthesis of platinum-coated Fe_3O_4 nanoparticles (Pt@FeNPs)

Platinum-coated FeNPs (Pt@FeNPs) was synthesized according to a previously published

method with slight modifications.[4] Typically, 5 mL aqueous solution of K_2PtCl_4 (20 mM) and Pluronic F127 (0.794 mM) was aged for 1 day under continuous stirring. 25 mg of FeNPs was added to the solution and sonicated for 10 min. Subsequently, 2.5 mL of ascorbic acid solution (0.2 M) was added as the reductant and sonicated for 30 min at 45 °C. The particles were then washed with MQ water for three times and dried under vacuum. Particles were grinded before further use.

S2.7 | Fabrication of hydrogel particles with various Pt@FeNPs localization

For the preparation of hydrogel micromotors, 10 mg mL^{-1} catalysts, i.e. Pt@FeNPs, were added to the inner phase. The other solutions were kept the same. To achieve well-dispersed inner solution, the Pt@FeNPs loaded inner solution was sonicated for at least 1 h before usage in microfluidics.

The localization of Pt@FeNPs in hydrogel particles was realized via the employment of a permanent NdFeB magnet (Company: Supermagnete, cube, side length: 10 mm, coating: Ni-Cu-Ni) during the collection and cross-linking of hydrogel particles. When no magnet was used, Pt@FeNPs were homogeneously distributed inside the hydrogel particles (Body). For head-shifted Pt@FeNPs hydrogel micromotors (Head), a cubic magnet with a surface field strength of 500 mT was placed 3 mm below the collection vial (1.5 mL mass vial) with 1.25 mL collection solution until the cross-linking was finished. For tail-shifted Pt@FeNPs hydrogel micromotors (Tail), the magnet was placed around 3 mm above the top of Eppendorf tube (0.5 mL) with 0.4 mL collection solution until the cross-linking was finished.

S2.8 | Inductively coupled plasma mass spectroscopy (ICP-MS) measurements

The percentage of Pt and Fe in Pt@FeNPs were determined by ICP-MS. Briefly, Pt@FeNPs were digested in aqua regia (3:1 of 37% HCl and 65% HNO_3; Note: Handle with care!) (0.05 mg mL^{-1}) overnight.[5] Subsequently, the digested nanoparticle solution was diluted 100 times with 0.1% HNO_3. The concentrations of Pt and Fe were measured by ICP-MS (Xseries, Thermo Fisher Scientific).

S2.9 | Characterization of surface and internal structures of hydrogel particles

The surface and internal structures of the blank and Pt@FeNP-encapsulated hydrogel particles were characterized by scanning electron microscopy (SEM) and cryo-scanning electron microscopy (cryo-SEM), respectively. For SEM, a few microliters of particle solution were

deposited on a flat copper plate or carbon-coated copper grids followed by drying overnight. The dried samples were then sputtered with palladium-gold (Cressington 208HR sputter coater) before characterization with SEM (JEOL 6330 field emission scanning electron microscope). For cryo-SEM, particle solution was rapidly frozen in liquid nitrogen and freeze fractured in the cooling pre-chamber of the microscope (JEOL 6330 field emission scanning electron microscope). The sample was subjected to palladium-gold sputtering before transferring to the chamber of the microscope, which was maintained at −120 °C.

S2.10 | Autonomous motion analysis

Autonomous movement of micromotors was studied under an optical microscope in 2.0%, 3.5%, 5.0% and 7.0% H_2O_2 (weight percentage), without any addition of surfactant. Typically, 2 mL H_2O_2 solution was added to a petri dish (3 cm in diameter). Recording immediately started upon the addition of 2 µL solution of micromotors to the H_2O_2 fuel.

The same procedures were performed for motion investigation in viscosity studies and in particle dimension studies.

Movies of the motion of micromotors were analyzed using MTrackJ for Fiji. Tracking was performed with efficient motors for 20 seconds.

S2.11 | Statistical analysis

Data analysis was performed with SPSS 29.0.1.0 (IBM Corp., USA). The results were expressed as means ± standard deviation (SD). Non-parametric Kruskal–Wallis tests with Bonferroni multiple comparison tests were used. A p value <0.05 was considered statistically significant.

S3 | Supplementary Figures

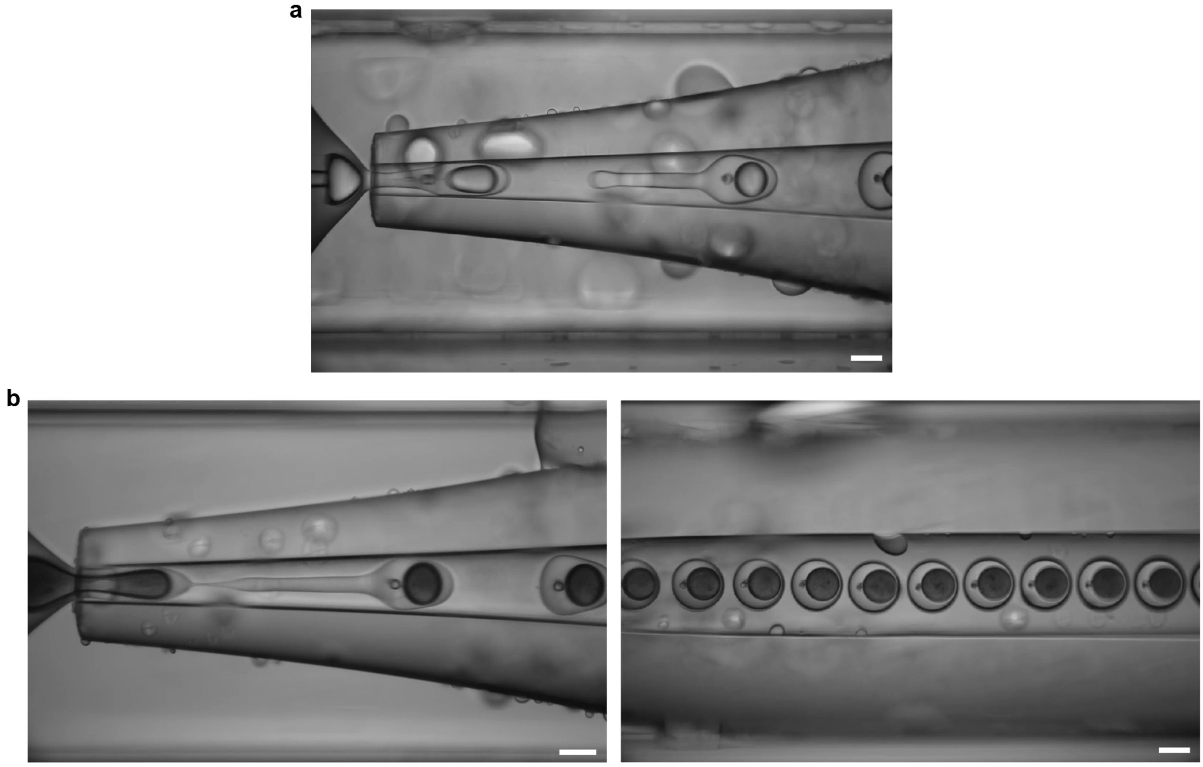

Figure S1. Optical images of double emulsion droplets in the microfluidic devices. (a) Blank alginate/mineral oil double emulsion droplets. (b) Alginate/mineral oil double emulsion droplets with 10 mg mL^{-1} Pt@FeNPs in the inner phase. Scale bars are 100 µm.

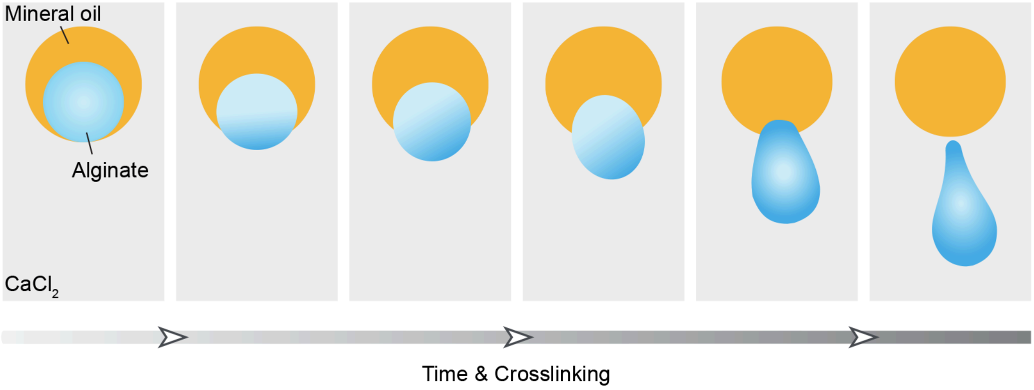

Figure S2. Time and cross-linking series of schematic images showing the separation of the inner alginate drop from the mineral oil shell. Adapted from ref. 1.

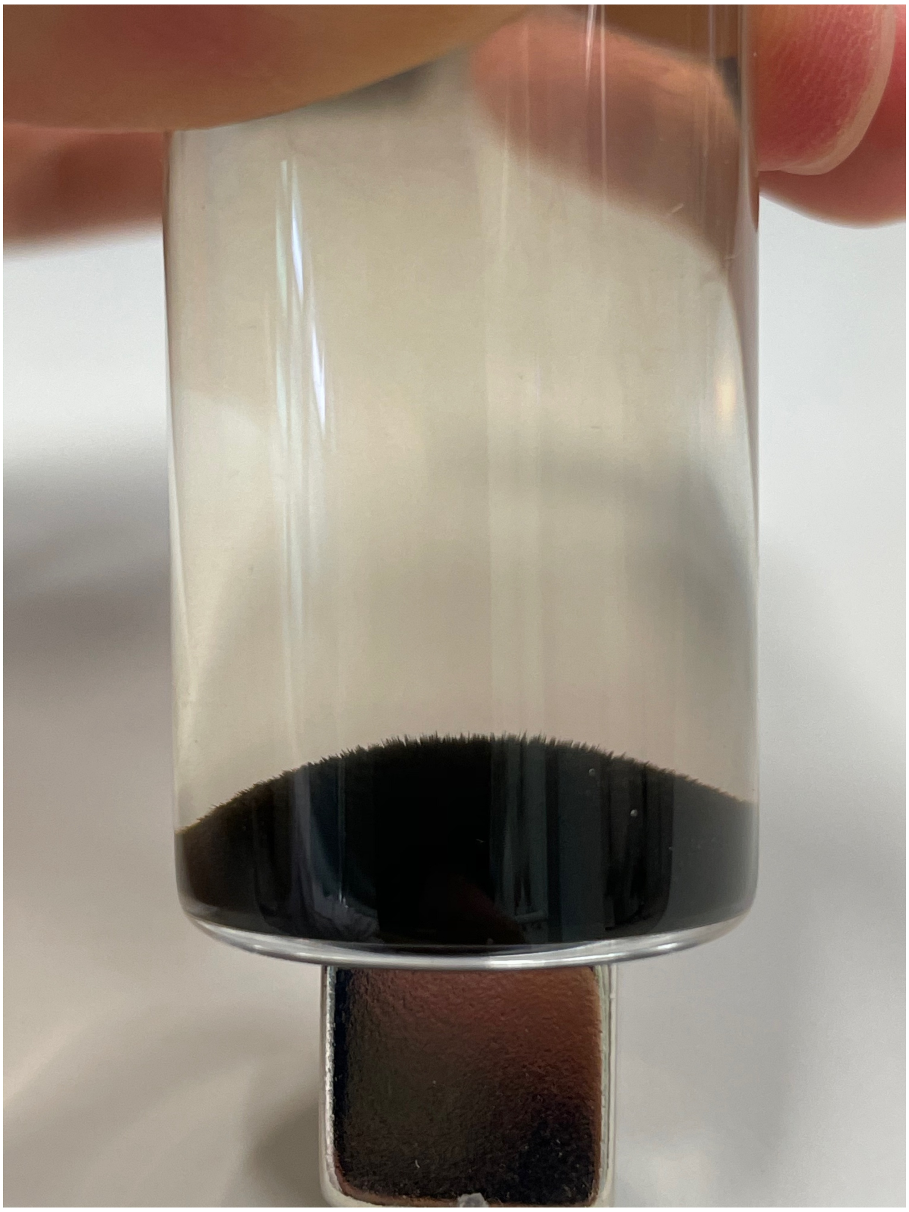

Figure S3. Digital image of Pt@Fe NPs in the presence of magnetic field.

Table S1. Viscosity of glycerol aqueous solutions[6]

Glycerol (wt.%)	Viscosity (mPa • s)
0	1.00
24	2.01
48	5.45
60	10.9

The viscosity of the in vivo fluid environment ranges from 1 to 10 mPa • s. Typically, the viscosity of blood plasma is approximately 2.2 mPa • s,[7] while human serum exhibits a viscosity of around 1.5−3 mPa • s.[8-9] The viscosity of gastric acid can be as high as ≈10 mPa • s.[10] To simulate the viscosity of in vivo environment, three different glycerol concentrations were selected to investigate the relationship between motion speed and viscosity (**Table S1**). The concentration of H_2O_2 fuel remained constant at 3.5% throughout the study.

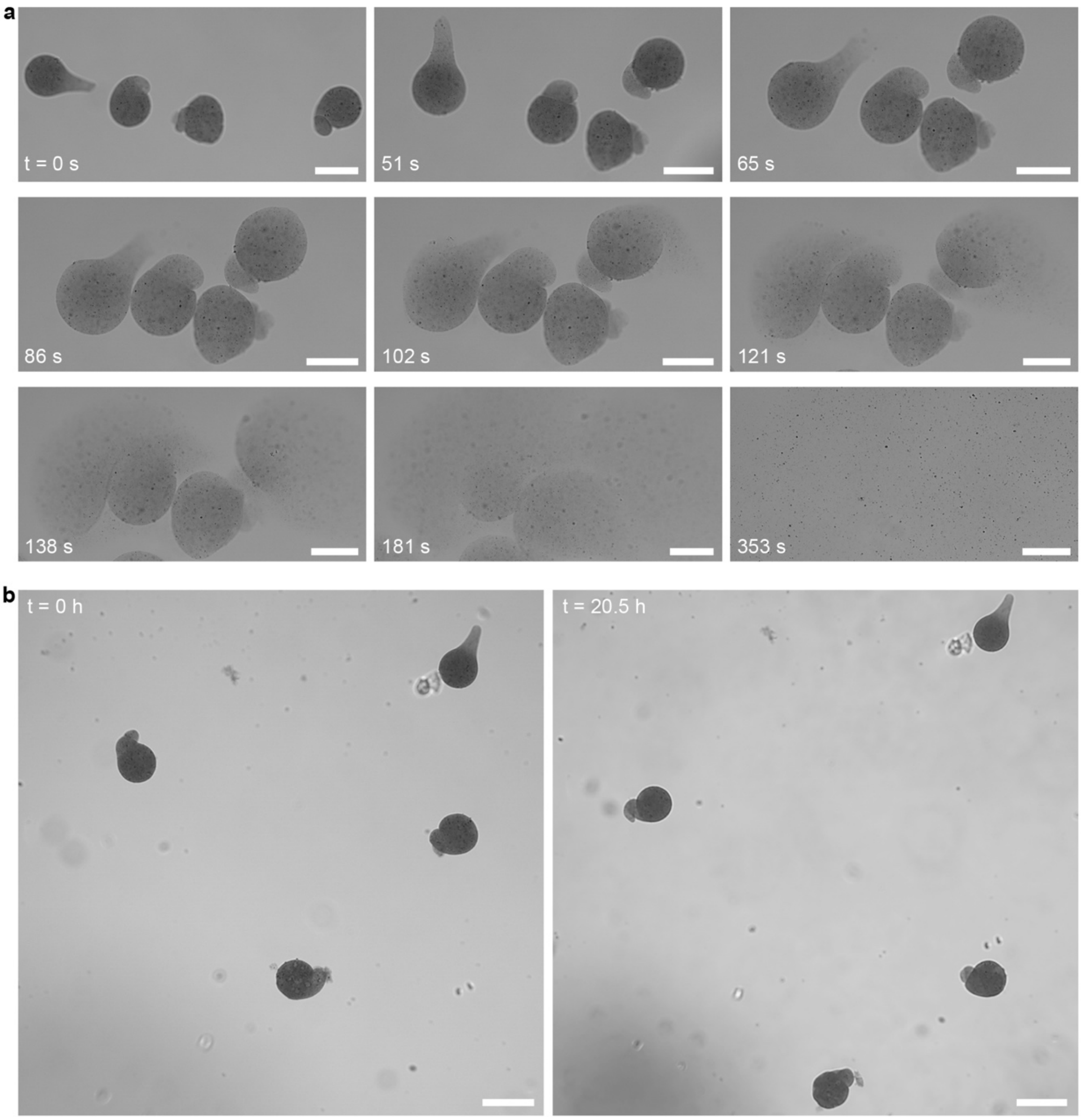

Figure S4. Degradation and stability of Body hydrogel micromotors. Time-lapse images of micromotors in (a) 0.1× PBS (pH 7.2) and (b) simulated gastric fluid (pH 1.1−1.3). Scale bars are 100 μm.

The Ca^{2+} crosslinked alginate hydrogel micromotors are sufficiently stable in the aqueous media for months (data not shown). When immersed in PBS medium at neutral pH of 7.2, ion-exchange process between Na^{+} and Ca^{2+} ions triggered the swelling and subsequent degradation of the particles (**Figure S4** and ref.11). On the other hand, when the particles were exposed to simulated gastric fluid with pH 1.1−1.3, the carboxylate ions of alginate underwent protonation, resulting in the formation of alginic acid. This process appears to hinder the

exchange between Na^+ and Ca^{2+} ions, leading to improved particle stability compared to that observed in the neutral PBS medium (**Figure S4**b).

S4 | References

(1) Martinez, C. J.; Kim, J. W.; Ye, C.; Ortiz, I.; Rowat, A. C.; Marquez, M.; Weitz, D. A microfluidic approach to encapsulate living cells in uniform alginate hydrogel microparticles. *Macromol. Biosci.* **2012**, *12*, 946–951.

(2) Vibhute, M. A.; Schaap, M. H.; Maas, R. J. M.; Nelissen, F. H. T.; Spruijt, E.; Heus, H. A.; Hansen, M. M. K.; Huck, W. T. S. Transcription and Translation in Cytomimetic Protocells Perform Most Efficiently at Distinct Macromolecular Crowding Conditions. *ACS Synth. Biol.* **2020**, *9*, 2797-2807.

(3) Hong, Y.; Shi, H.; Shu, X.; Zheng, Y.; Zhang, Y.; Wu, Y. Controlled synthesis of hollow magnetic Fe 3 O 4 nanospheres: Effect of the cooling rate. *Particuology* **2017**, *33*, 24-28.

(4) Wilson, D. A.; Nolte, R. J. M.; van Hest, J. C. M. Autonomous movement of platinum-loaded stomatocytes. *Nat. Chem.* **2012**, *4*, 268-274.

(5) Sápi, A.; Kéri, A.; Kálomista, I.; Dobó, D. G.; Ákos Szamosvölgyi, Á. S.; Juhász, K. L.; Ákos Kukovecz, Á. K.; Kónya, Z.; Galbács, G. Determination of the platinum concentration of a Pt/silica nanocomposite decorated with ultra small Pt nanoparticles using single particle inductively coupled plasma mass spectrometry. *J. Anal. At. Spectrom.* **2017**, *32*, 996-1003.

(6) Haynes, W. M., *CRC handbook of chemistry and physics*. 95th ed. ed.; CRC Press: Boca Raton, FL: 2014; p 5-131.

(7) Mou, F.; Chen, C.; Zhong, Q.; Yin, Y.; Ma, H.; Guan, J. Autonomous Motion and Temperature-Controlled Drug Delivery of Mg/Pt-Poly(N-isopropylacrylamide) Janus Micromotors Driven by Simulated Body Fluid and Blood Plasma. *ACS Appl. Mater. Interfaces* **2014**, *6*, 9897-9903.

(8) Gao, W.; Sattayasamitsathit, S.; Orozco, J.; Wang, J. Highly efficient catalytic microengines: template electrosynthesis of polyaniline/platinum microtubes. *J. Am. Chem. Soc.* **2011**, *133*, 11862-11864.

(9) Gao, W.; Sattayasamitsathit, S.; Orozco, J.; Wang, J. Efficient bubble propulsion of polymer-based microengines in real-life environments. *Nanoscale* **2013**, *5*, 8909–8914.

(10) Pedersen, P. B.; Vilmann, P.; Bar-Shalom, D.; Mullertz, A.; Baldursdottir, S. Characterization of fasted human gastric fluid for relevant rheological parameters and gastric lipase activities. *Eur. J. Pharm. Biopharm.* **2013**, *85*, 958-965.

(11) Bajpai, S. K.; Sharma, S. Investigation of swelling/degradation behaviour of alginate beads crosslinked with Ca^{2+} and Ba^{2+} ions. *React. Funct. Polym.* **2004**, *59*, 129-140.
